# Case Report: Detection of SARS-CoV-2 From Cerebrospinal Fluid in a 34-Month-Old Child With Encephalitis

**DOI:** 10.3389/fped.2021.565778

**Published:** 2021-04-20

**Authors:** Fatemeh Cheraghali, Alireza Tahamtan, Seyed Ahmad Hosseini, Mohammad Hadi Gharib, Abdolvahab Moradi, Hadi Razavi Nikoo, Alijan Tabarraei

**Affiliations:** ^1^Department of Pediatrics, School of Medicine, Taleghani Children's Hospital, Golestan University of Medical Sciences, Gorgan, Iran; ^2^Infectious Diseases Research Centre, Golestan University of Medical Sciences, Gorgan, Iran; ^3^Department of Microbiology, School of Medicine, Golestan University of Medical Sciences, Gorgan, Iran; ^4^Department of Radiology, School of Medicine, 5th Azar Hospital, Golestan University of Medical Sciences, Gorgan, Iran

**Keywords:** SARS-CoV-2, COVID-19, pediatrics, CSF, case report

## Abstract

Novel coronavirus (severe acute respiratory syndrome-coronavirus-2: SARS-CoV-2), which arose from Wuhan, China, has rapidly spread to other countries and developed into a pandemic. Although the respiratory manifestations of SARS-CoV-2 are well-documented, there is a considerable challenge regarding the direct and/or indirect infection in other organs. Several preliminary reports confirmed neurological manifestations in the SARS-CoV-2-infected patients. Here, we report the detection of SARS-CoV-2 from the nasopharyngeal swab and cerebrospinal fluid (CSF) in a 34-month-old child with encephalitis. This finding expands the spectrum of the neurological manifestations associated with SARS-CoV-2 infection.

## Introduction

Coronavirus disease 2019 (COVID-19) is a recently emerged pandemic disease caused by the novel coronavirus, severe acute respiratory syndrome coronavirus 2 (SARS-CoV-2) (1). Although the respiratory manifestations of SARS-CoV-2 are well-recognized, there is a considerable challenge regarding the direct and/or indirect infection in other organs (2). According to the recent reports, neurological manifestations may be the presenting feature of COVID-19 (3, 4). However, the exact route of entry to the central nerve system (CNS) and pathophysiologic mechanism behind the neurological manifestations of the SARS-CoV-2 infection are unclear. Research is continuing to uncover the different aspects of SARS-CoV-2 infection. Here, we describe a child in Iran with encephalitis that was detected positive for the SARS-CoV-2 genome from the nasopharyngeal swab and cerebrospinal fluid (CSF) by real-time reverse transcriptase-PCR (RT-PCR). This case represents that clinicians should be aware of different neurological presentations of COVID-19 and should not underestimate any neurological findings during the current pandemic.

## Case Description

**Figure 1:**
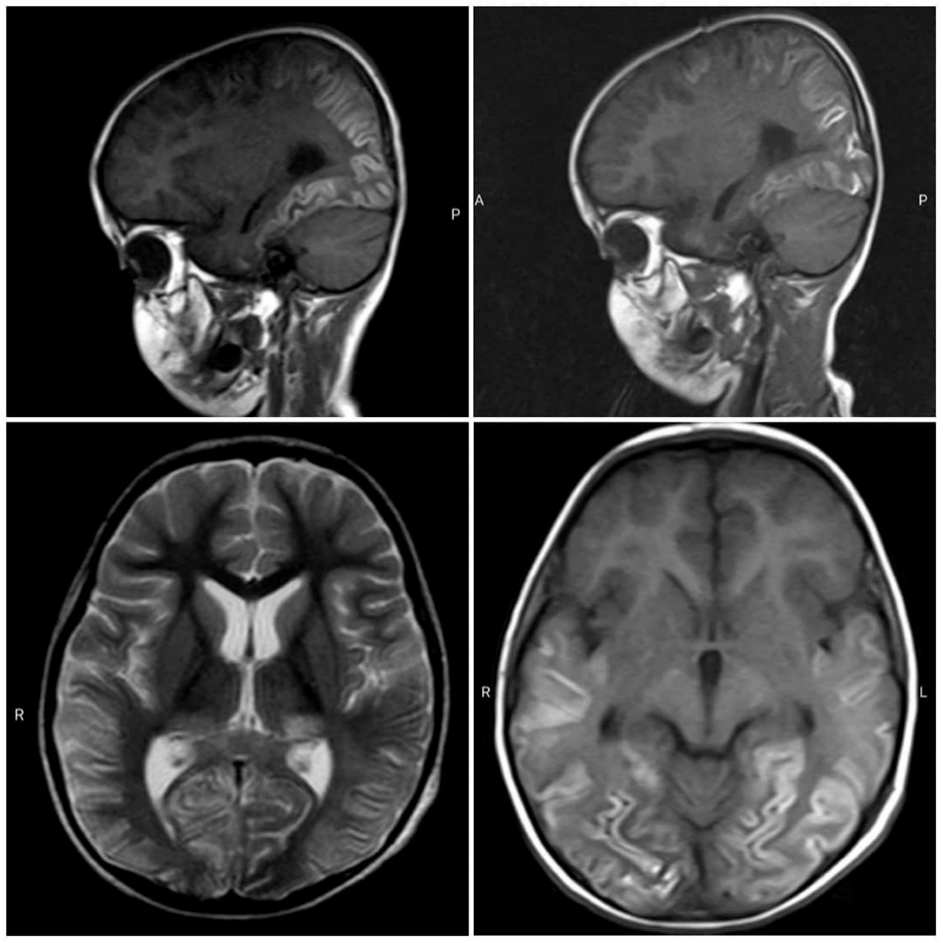
MRI obtained from brain. Images of brain MRI proposed mostly symmetric, cortical, and juxta-cortical high T1 and T2 signal abnormality, in bilateral parieto-occipital lobes.

**Figure 2:**
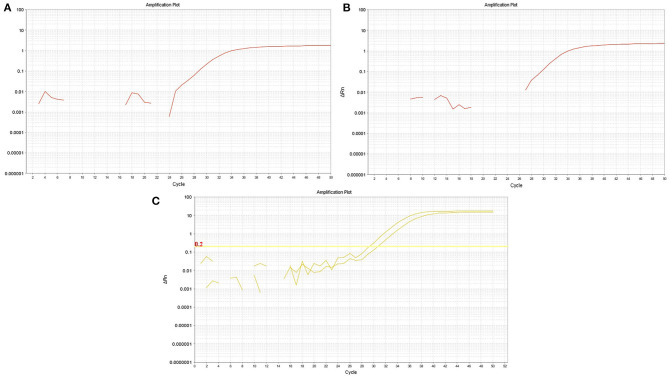
Real-time RT-PCR graph. Real-time RT-PCR showed the presence of SARS-CoV-2 genome in both the CSF **(A)** and respiratory **(B)** samples, targeting the SARS-CoV-2 E gene. The positive CSF result was confirmed by another real-time RT-PCR test targeting the SARS-CoV-2N and ORF1ab genes **(C)**.

**Figure 3:**
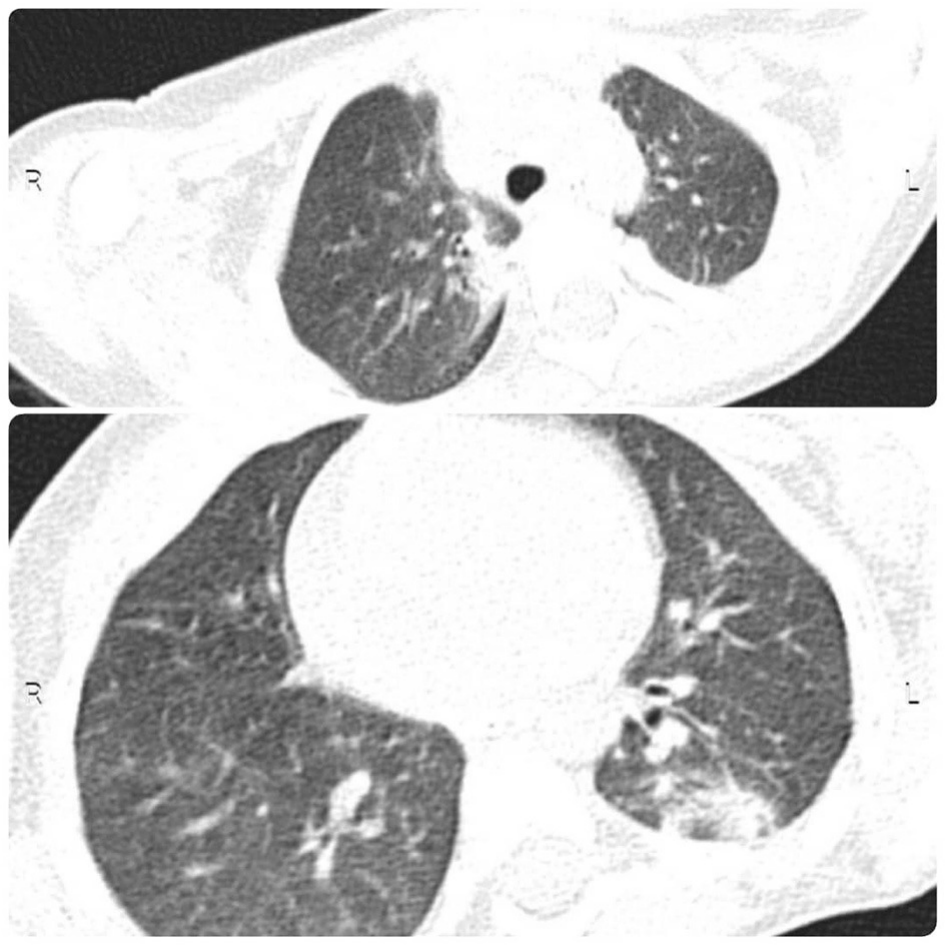
CT obtained from chest. Chest CT showed underlying bronchopneumonic lung involvement.

A 34-month-old child was admitted to the Tamin Ejtemae Hospital in Gonbad city, north of Iran, on March 9, 2020 because of a 4-day history of fever, seizure, and upward gaze. Before the onset of mentioned symptoms, the child was healthy with normal nerve function and had normal development. A medical history of febrile convulsion was observed in his sister. At admission, the main laboratory findings were increased in white blood cell counts (WBC), elevated level of blood sugar, and positive in C-reactive protein (CRP) test. All laboratory characteristics are shown in Table 1. After 4 days, the child was referred to the Intensive Care Unit (ICU) of Taleghani Hospital in Gonbad city due to fever, repeated generalized tonic-clonic seizures, and loss of consciousness. Lumbar puncture (LP) was performed, and CSF appeared clear with normal protein and glucose level and negative for bacterial growth (Table 2). The most relevant laboratory findings included elevated levels of aspartate aminotransferase (AST), alanine aminotransferase (ALT), erythrocyte sedimentation rate (ESR), lactic acid dehydrogenase (LDH), and an increase in WBC counts (Table 1). With these clinical and laboratory characteristics, the attended physician started treatment with Meropenem, Vancomycin, Acyclovir, and anti-seizure drugs. Due to the recurrent seizures and Glasgow Coma Scale of 5–6, he was transferred to the pediatric ICU (PICU) of the Taleghani Children Hospital in Gorgan city on March 26, 2020. He was in a decerebrate posture at admission, his pupils were mydriatic with maximum size but reactive, and his limbs were spastic. The Babinski sign could not be evaluated, and he had a partial response to painful stimuli. The patient had repeated seizures during hospitalization in the PICU. The main laboratory findings were AST of 169 U/L, ALT of 173 U/L, WBC of 23,100 cells/μL, and platelets of 727,000 cells/μl (Table 1). Another LP did not reveal any evidences of CNS infection (Table 2). His brain magnetic resonance imaging (MRI) findings proposed mostly symmetric, cortical, and juxta-cortical high T1 and T2 signal abnormality, in bilateral parieto-occipital lobes. A few differential diagnoses were proposed; however, it was mostly compatible with viral encephalitis, with possible parenchymal hemorrhagic components (Figure 1). Follow-up blood tests revealed AST of 133 U/L, ALT of 138 U/L, lactate of 38 mg/dl, creatine phosphokinase (CPK) of 1,775 U/L WBC of 16,900, and LDH of 2,826 U/L (Table 1). The molecular test for Herpes simplex virus 1 and 2 was negative. Given the COVID-19 pandemic, SARS-CoV-2 infection was suspected, and the attended physician ordered testing. Viral RNA was extracted from the collected nasopharyngeal swab, CSF, and blood samples and analyzed by real-time RT-PCR assay targeting the envelope (E) gene. The test showed that SARS-CoV-2 RNA was present in both the nasopharyngeal swab and CSF samples with the same cycle threshold (Figure 2). The positive CSF result was confirmed by another real-time RT-PCR targeting the nucleoprotein (N) and ORF1ab genes. After confirmation of COVID-19 by real-time RT-PCR, bilateral peripheral consolidations in RUL and superior segment of LLL in a spinal chest CT scan showed underlying bronchopneumonic lung involvement (Figure 3). At admission to Taleghani Children Hospital in Gorgan city, treatment was started with dexamethasone and wide-spectrum antibiotics, but after SARS-CoV-2 detection, prescription changed to hydroxychloroquine, azithromycin, and intravenous immunoglobulin (IVIG), and after 5 days, caltra was used. After the mentioned treatment, the patient became ventilator independent and discharged. His oxygen saturation improved, and well-controlled seizure was achieved, but he was still in a decerebrate posture. Gradually, the state of consciousness improved, but he had no communication with the environment, and his limbs were still spastic. With the diagnosis of vegetative state, the patient was discharged, and necessary instructions were trained. At the moment, he is fed with nasogastric tube, his communication with the environment is not good, and he is not able to speak and make constant eye contact.

**Table 1:** Patient laboratory characteristics.

**Test**	**9 Mar**	**12 Mar**	**14 Mar**	**16 Mar**	**18 Mar**	**20 Mar**	**22 Mar**	**25 Mar**	**26 Mar**	**29 Mar**	**31 Mar**
WBC, cells/μl	14,700	9,300	7,700	12,300	8,400	12,900	27,800	23,600	23,100	14,200	16,900
RBC, Mill/mm3	5.4	4.5	4.85	4.97	6.3	5.9	5.5	5.2	5.41	5.16	4.89
Hb, g/dl	8.8	7.5	7.5	8.1	14	12.6	12.5	11.4	12.3	11.5	11.5
Hct, %	30.6	26.5	27.3	27	41	38.5	37.6	34.7	36.7	35.2	33.6
MCV, fl	55.74	58.76	56.3	54.33	64.8	65.14	68.36	66.48	67.44	68.22	68.71
MCH, pg	16.03	16.63	15.5	16.3	22.15	21.32	22.73	21.84	21.34	22.29	23.52
MCHC, g/dl	28.76	28.3	27.5	30	34.15	32.73	33.24	32.85	33.51	32.6	34.23
PLT, k/μL	315,000	242,000	282,000	300,000	280,000		435,000	882,000	727,000	445,000	226,000
Poly, %	78	65	82				78	80	67	65	60
Lymph, %	20	35	16				20	20	29	35	37
Mono	1								2		1
Eos	1		2				2		2		2
RDW	17.3			17	30.1	30		29.4	28.6	28.9	29.8
Hypo			+1			1			+1		+1
Aniso			+1			+1			+1		+1
Micro									+1		+1
ESR, mm/h		26		54	20	20	14	17	15		18
PT, sec			13							15	15
INR			1				1.1			1.2	1.2
PTT, sec			31							32	35
AST, U/L			81		183	442	128	111	169	154	133
ALT, U/L			14		45	414	346	178	173	111	138
ALP, U/L					347	509		420	348	385	390
Calcium, mg/dl	8.5		9.4	8.4	8.9	9.5		10	9		
Phosphorus, mg/dl					2.6				2.3		
Mg, mEg/L				2.2	2.2			1.9	1.6		
CPK, U/L	115			214	166						1,775
LDH, U/L	453			1,842	3,496						2,826
Ammoniac									90		
Lactat, mg/dl									19	31	38
Bun, mg/dl	12		4		5	5	7	7	7	7	9
Creatinine, mg/dl	0.4		0.6		0.4	0.4	0.42	0.4	0.5	0.5	0.55
Na, mg/dl	129		124	129	129	29	135	134	142	142	143
K, mg/dl	3.4		3.9	3.1	4.4	4.2	5.3	6.5	4.8	4.5	5
Total protein, g/dl						8.4		9.5		6	5.5
Alb, g/dl						3.5		4.3		3.6	3.7
Troponin											negative
CRP	+1	+1	+1	negative	negative	+1			negative	+1	+1
BS	218		113		139			98			

**Table 2:** Patient CSF analysis.

**Test**	**Mar 12**	**Mar 29**	**Mar 31**
Color	Colorless	Colorless	Colorless
Appearance	Clear	Clear	Clear
WBC	0 cells/μl	2 cells/ μl	1
RBC	100 cells/ μl	5 cells/ μl	8
Glucose	100 mg/dl	65 mg/dl	45 mg/dl
Protein	15 mg/dl	39 mg/dl	20 mg/dl
LDH	47 U/L		
Lactat		25 mg/dl	23 mg/dl
Bacteriology culture	No growth	No growth after 24, 48, and 72 h	

## Discussion

While neurological complications such as seizures, encephalopathy, and encephalitis are rare in respiratory viral infections, neurological manifestations have been suggested as a presenting feature of COVID-19 (3, 4). Importantly, the SARS-CoV-2 genome has been detected in CSF and brain tissue of COVID-19 patients, and viral infection of the neurons was confirmed (5–9). The CSF analysis in this patient was normal; therefore, it is unclear how the virus crossed the blood–brain barrier (BBB) and caused such neurological complications. It is unclear whether SARS-CoV-2 can transmit directly to the CNS or the virus infects resident, infiltrating, and circulating immune cells, in which the circulating immune cells carry the virus to the CNS. It is also unclear how the neurologic manifestations caused by direct replication of SARS-CoV-2 in the CNS or the virus-induced inflammation promoted neuronal injury. However, there is evidence that SARS-CoV-2 infects the brain choroid plexus and disrupts the BBB (10). It is also important to note that Alexopoulos et al. have recently detected high-titer anti-SARS-CoV-2 antibodies in the CSF of comatose or encephalopathic patients demonstrating intrathecal IgG synthesis or BBB disruption (11). Other studies have also hypothesized that the virus can damage the nervous system through several mechanisms, including direct infection, hypoxia, ACE-2, and immune-mediated (12).

Although the COVID-19 can affect all age groups, the disease is usually milder in children than in adults and may be accompanied by non-specific symptoms (13). Notably, the patient chest CT findings are not a classical appearance of COVID-19 lung pneumonia, because it is usually anticipated in adults. Albeit, chest CT findings in children with COVID-19 are mostly subtle and non-specific, it is also good to consider that this case had several days history of hospitalization before undergoing chest CT, and hence, the possibility of hospital-acquired pulmonary superinfection is also a plausible explanation for the pulmonary findings. To the best of our knowledge, this is the first report of encephalitis associated with COVID-19 in pediatrics that highlights the importance of consideration of neurological complications as unknown signs of COVID-19. Hence, more cases with precise reports and further investigation would be required to determine the neuroinvasive potential of SARS-CoV-2, possible entry route, and explain the mechanisms contributing to the pathophysiology of neurological manifestations associated with COVID-19. As the number of patients with COVID-19 increases worldwide, clinicians should be aware of different neurological presentations of COVID-19 and should not underestimate any neurological findings, especially in pediatrics.

## Data Availability Statement

The original contributions presented in the study are included in the article/supplementary material, further inquiries can be directed to the corresponding author/s.

## Ethics Statement

Ethical review and approval was not required for the study on human participants in accordance with the local legislation and institutional requirements. Written informed consent to participate in this study was provided by the participants' legal guardian/next of kin. Written informed consent was obtained from the individual(s), and minor(s)' legal guardian/next of kin, for the publication of any potentially identifiable images or data included in this article.

## Author Contributions

FC and ATah conceptualized and designed the study, drafted the initial manuscript, and reviewed and revised the manuscript. ATab designed the data collection instruments, coordinated and supervised data collection, and critically reviewed the manuscript. SH, MG, AM, and HR collected data, carried out the initial analyses, and reviewed and revised the manuscript. All authors approved the final manuscript as submitted and agree to be accountable for all aspects of the work.

## Conflict of Interest

The authors declare that the research was conducted in the absence of any commercial or financial relationships that could be construed as a potential conflict of interest.
